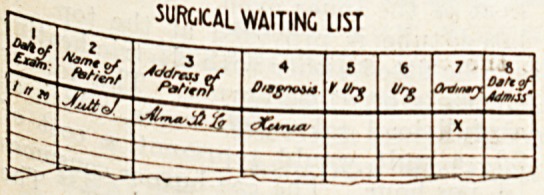# The Institutional Worker

**Published:** 1920-11-13

**Authors:** 


### THE INSTITUTIONAL WORKER

**Being a Special Supplement to "The Hospital"**

### OUR BUREAU OF INFORMATION.

### Rules for Correspondents

1. Every letter must be accompanied by the coupon to be cut from the back cover (inside page) of The Hospital, current issue, and must contain the name and address of the correspondent with pseudonym for publication if desired. Replies by post cannot be given save under exceptional circumstances at the Editor's discretion.

2. Letters from Approved Homes in reply to special needs published in the Bureau should state terms and full particulars, and be sent prepaid under cover to the Editor of the Bureau with name written across coupon for identification.

3. Proprietors of Homes which have not yet been entered on the List of Approved Homes, but have spare accommodation likely to suit special needs, are invited to write for an application form for registration. The fee for registration, which includes two announcements of the Home in the Bureau and other privileges, is 10s.

4. All communications to be addressed to the Editor of The Hospital, 28 Southampton Street, Strand. London, W.C. 2, and marked "Bureau of Information."

### INSTITUTION FACTS AND FIGURES.

### The Question Box.

#### PATIENTS' WAITING-LISTS.

The question was:

**Describe a simple method of recording names of patients awaiting admission, showing at a glance their proper turn for admission, having regard both to period of waiting and the medical urgency of their respective cases.**

**G. W. replies:—**

Institutions differ so much in the method of the allocation of the beds to the various physicians or surgeons giving their services to the institution, that it is impossible to outline a system that will be suitable to all. I shall confine my explanation to the method I have had experience with—*i.e.*, where a fixed number of beds is allocated to each physician or surgeon. It is very necessary to emphasise that all the medical and surgical staff of the institution must work in close co-operation in order to render the undermentioned method of practical value.

Each of the staff has his own waiting-book, and is responsible for the rotation of admission of all his waiting cases.

The following diagram presents a page from one of the surgeon's waiting-book:

SURGICAL WAITING LIST

| 1 Date of Exam: | 2 Name of Patient | 3 Address of Patient | 4 Diagnosis. | 5 V Urg | 6 Urg | 7 Ordinary | 8 Date of Admiss[n] |
|---|---|---|---|---|---|---|---|
| 1 11 20 | Nutt J. | Alma St. Lo | Hernia | | | X | |

Columns 1, 2, 3, and 4 are self-explanatory.

Column 5.—Crosses should be placed in this column in respect of all cases which are of such a nature as to call for quick admission while they are not of sufficient urgency to come under the category of emergencies. These latter should be sent to the receiving-room for immediate admission or whatever method is adopted by the institution concerned.

Column 6.—For cases which are not quite so urgent but are more urgent than those to go in Column 7.

Column 7.—For ordinary cases such as hernia, etc., or those cases which can be temporarily relieved by the provision of a surgical appliance.

The physician or surgeon works his book as follows.

Column 5 has priority over all cases. Column 6 comes next, and Column 7 is disposed of whenever there are any beds left vacant after Columns 5 and 6 have had consideration. Column 8 should be filled in when the notice of a vacant bed is sent to the patient. If that patient refuses or desires to postpone admission, then the name must be re-entered and the patient must await the next turn just as if the first entry had never been made.

It can be seen how necessary a thing is co-operation between the various members of the professional staff. One surgeon may have no Column 5 cases on his list, and another may have more of these cases than he can find beds for. Again, one physician may have exhausted his waiting-list and have a vacant bed while his colleagues may have urgent cases awaiting admission. With genial co-operation these difficulties are easily overcome. Then if any hitch occurs it can be referred to the Medical Committee of the institution, which is generally composed of all the honorary staff.

The above is a very simple method, although, of course, if it is possible to have all cases placed on the same list, irrespective of who is in charge, it would be much better. With this method the selection of cases would either have to be done by the Medical Committee or by an appointed member of the staff. Neither of these methods gives entire satisfaction, in the first case, because other and more important business occupies the time of the Medical Committee, whilst the second has its obvious objections.

### To Wash Feather Pillows.

*The Modern Hospital* (Chicago) publishes a suggestion for washing feather pillows without removing the ticking, which, it states, has proved practical. Care should be observed that the washing machine is not too amply filled with the pillows, and that only about 4 inches of water about 80° F., with a small amount of washing soda, is admitted. After allowing the machine to run for ten minutes the pillows should be rinsed for about five minutes in the same depth of water and the same temperature. For the third washing the temperature of the water should be 100°, the amount is still the same, and enough soap should be added to make good suds. The machine should then run for fifteen minutes. The next steps are a fourth and fifth rinse, for each of which the same amount of water is used, the temperature in each case being 80°. The pillows should then be extracted in the usual way, and if available a heated tumbler should be used for drying, the temperature of which should not exceed 100°. If a tumbler is not available the pillows should be dried in the dry-room, care being taken that the heat is not excessive, or otherwise the feathers will be spoilt.

Soft water is very desirable in all the washing processes, and another necessary precaution is to repair all weak places in the ticking before the washing is begun.

### Question for November.

### A NURSES' LIBRARY.

Describe fully the most practical way of forming and running a nurses' library suitable for probationers, staff nurses, and sisters. The description should include an outline of necessary rules, the administration, the selection of books, finance, and a method for keeping the library up to date and disposing of old books.

**(A minimum payment of 10s. 6d. will be made for each published answer.)**

RULES.

The following rules must be observed :—

1. Contributions must be written on one side of the paper. Conciseness and terseness are desirable features. The MS. must bear the name and address of the sender and be accompanied by coupon to be cut from the back cover (inside page) of the current issue of THE HOSPITAL. A pseudonym must be chosen if the name is not to be published.

2. Contributions must be addressed to the Editor of THE HOSPITAL Institutional Worker Supplement, 28 & 29 Southampton Street, Strand, London, W.C. 2, should reach him before the end of the current month, and be marked in the left-hand corner "Facts and Figures."

### QUESTIONS INVITED.

IN connection with our Question Box, we offer every month five shillings for the best question sent in for consideration. The questions may be on any institutional subject and concern any department, from the secretary's to the porter's, or the matron's to the domestic staff; the one essential is that they must be practical and deal with points that have a definite relation to hospital or institutional administration, upkeep, finance, or management. Points relating to artificers' work, housekeeping, the laundry, out-patients, and other practical matters will be welcome. It is hoped that thus, when difficult or doubtful points arise in the course of their work, institutional workers will be encouraged to put them in the form of a question and send them to the Editor, THE HOSPITAL Bureau of Information, 28 Southampton Street, Strand, W.C. 2, marked "Question Box."

The best questions will be published in due course and our readers will be given the opportunity of answering them. Thus all institutional workers may be able to co-operate with the view of helping the work and smoothing the difficulties of each other. In short, we want the Question Box of the *Institutional Worker* to be freely used by every worker habitually.

### ENQUIRIES AND ANSWERS.

### *EMPLOYMENT AND TRAINING.*

#### Training for Disabled Nurse.

WE suggest that you write to the Ministry of Labour, St. Ermin's Hotel, Westminster, S.W. The Ministry gives suitable training to nurses disabled in the war.—DISABLED NURSE.

#### Visiting Nurse.

THE Committee of the Nurses' Co-operation, 22 Langham Street, W., have recently decided to supply fully-trained nurses for daily visiting in addition to their regular staff, and we suggest that you write to the matron and inquire if she has a vacancy on her staff for a visiting nurse. Enclose stamped envelope for reply.—NURSE M.

#### Fever Training.

WRITE to the Clerk of the Metropolitan Asylums Board (Dept. 9), Embankment, E.C. 4, and inquire if there are any vacancies in their group of hospitals. Probationers receive a salary at the rate of £47 to £49 per annum with board, lodging, washing, and uniform. A 50-hour working week is in operation at all their hospitals, and extra pay is given at special rates for any time worked in excess of the fifty hours per week.—DORCAS.

### *MISCELLANEOUS.*

#### Electrical Instrument for the Deaf.

MR. W. H. PETTIFOR, of 11 Victoria Street, Westminster, S.W. 1, supplies an excellent "electrical aid" for the deaf. The "aid" is composed of three parts: a receiver, or ear-piece; a transmitter, which is worn on the dress or placed on the table; and a small dry battery, which is carried in the pocket or placed on the table as desired. The three parts are connected by means of an insulated flexible conductor. The receiver may be held to the ear by means of a head-band passed underneath the hair; a man may wear the transmitter attached to the battery in the top waistcoat pocket, passing the receiver cord round the back of the neck under the coat, the receiver being placed in the other waistcoat pocket when not in use. A lady may wear the transmitter under her blouse, the battery being carried in a bag attached to her waist-belt. It is necessary to be tested for such an instrument before buying one. We suggest that you write to Mr. Pettifor for full particulars. The price of the "aid" is £6 6s.—K. B.

#### Electric Sterilizer.

CONRAD H. PINCHES is the sole agent in England for the Pelton Sterilizer supplied by the Pelton and Crane Company, Detroit, Michigan. The sterilizer is entirely constructed without solder, the boiler being of cast copper alloy "held in a shell of polished nickel brass, which tends to prevent radiation of heat. The heating coil is of the flat iron type held tightly against the bottom of the cast-iron boiler, which prevents waste of electricity. A great advantage is that it is impossible to burn it out. Dry sterilization can be accurately obtained. The size is 11 inches long, 5 inches wide, and 3 inches deep. Write to the agent at 13 Poland Street, London, W. 1, for price, etc.—ENQUIRER.

#### Comprehensive Work on Hygiene.

YOU should address yourself to the Secretary of the Royal Sanitary Institute, 90 Buckingham Palace Road, S.W., and ask him to suggest the best book for you to study hygiene. Enclose a stamped addressed envelope for reply.—F. C. S.

#### Diagnostic Apparatus.

A SIMPLE colorimeter made on the lines suggested by Dr. Cammidge is manufactured by Messrs. Hawksley & Sons, of 357 Oxford Street, London, W. 1. The price is £2 2s. The same firm also make an exceedingly convenient and reliable sphygnomanometer. The apparatus comprises a pressure gauge resembling an aneroid barometer, the dial of which (only 2 inches in diameter) is graduated in millimetres of mercury by comparison with a "standard" mercurial manometer, and registers from Zero to 300 mm., a broad bandage armlet, the rubber bag of which measures 13½ by 4½ inches, and a metal inflating pump with release screw. The price complete in roll-up case is £4 4s.—M. D.

#### Insulated Heat-Saving Oven.

AN insulated oven which must commend itself to all interested in fuel economy is made by Leoline Edwards, of St. Margaret's Road, Twickenham. Somewhat more costly than the ordinary oven, because of the material and labour involved in its construction, it is more than worth the additional outlay involved. This oven consists practically of two ovens, one within the other, the space between being packed or insulated with a fireproof non-conducting material which conserves the heat of the inner oven. A small ventilated tube is provided at the top. It can be fitted either with electric heating elements or a gas-burner. The former at full load takes 500 watts, which at 2d. a unit would represent a cost of 1d. per hour. The gas-burner consumes between 3 and 4 c. ft. per hour. The heating device can, if desired, be removed and the oven used as a hay-box. The price ranges from £7 13s. upwards.—A. T. C.

**The Editor will be glad to receive correspondence and to consider contributions upon all subjects relating to institutional work which affect the welfare of institutional workers.**

## Figures and Tables

**Figure f1:**